# Cyclin-Dependent Kinase 5 Regulatory Subunit Associated Protein 3: Potential Functions and Implications for Development and Disease

**DOI:** 10.3389/fonc.2021.760429

**Published:** 2021-10-14

**Authors:** Linna Sheng, Jiaxuan Li, Shengfang Rao, Zhijun Yang, Yonghong Huang

**Affiliations:** ^1^ Department of Pathophysiology, Basic Medical College of Nanchang University, Nanchang, China; ^2^ Graduate College of Nanchang University, Nanchang, China; ^3^ Nanchang Joint Program, Queen Mary School, Nanchang University, Nanchang, China; ^4^ Department of Nuclear Medicine, Nanchang University Hospital, Nanchang, China

**Keywords:** CDK5RAP3, C53, LZAP, embryogenesis, tissue development, tumorigenesis

## Abstract

Cyclin-dependent kinase 5 (CDK5) regulatory subunit associated protein 3 (CDK5RAP3, also named as C53 or LZAP) was initially identified as a binding protein of CDK5 activator p35. To date, CDK5RAP3 has been reported to interact with a range of proteins involved in cellular events ranging from cell cycle, apoptosis, and invasion to UFMylation modification and endoplasmic reticulum stress. Owing to its crucial roles in cellular processes, CDK5RAP3 is demonstrated to be not only an active participant in embryonic and mammalian tissue development, but also a key regulator in the onset and progress of human cancers such as head and neck squamous cell carcinoma, gastric cancer, hepatocellular cancer, lung cancer, kidney cancer and breast cancer. Notwithstanding, the detailed function of CDK5RAP3 and its mechanism remain poorly defined. Here, we briefly described a history of the discovery of CDK5RAP3, and systematically overviewed its gene structural and distribution features. We also focused on the known functions of this protein and its implications for embryogenesis and tissue development, as well as diseases especially carcinoma. This review may facilitate to understand the molecular and functional basis of CDK5RAP3 and its association with development and disease, and provide a reasonable idea for novel therapeutic opportunities targeting CDK5RAP3.

## Introduction

More than 20 years ago, Ching et al., initially isolated three novel binding partners of cyclin-dependent kinase 5 (CDK5) activator p35 from rat brain cDNA library by using the yeast two-hybrid screen assay (1). Among the three proteins, the protein with a molecular mass of 57 kDa was designated as C53, and later also denoted as CDK5 regulatory subunit associated protein 3 (CDK5RAP3) or LXXLL/leucine-zipper-containing alternative reading frame (ARF)-binding protein (LZAP) (2). CDK5RAP3 appears to be highly conserved in vertebrates, invertebrates and plants but not in yeast and bacteria (3). Moreover, CDK5RAP3 is widely expressed in human tissues and broadly located in subcellular compartments. Intriguingly, CDK5RAP3 does not have a known enzymatic domain or other well-described functional motifs. Recent research has indicated that CDK5RAP3 may perform its function possibly through interactions with its target proteins. To date, CDK5RAP3 has been found to interact with a variety of proteins and the details of its partners are shown in **Table 1**.

**Table 1:** Overview on identified interacting proteins of CDK5RAP3.

Target protein	Method	References
p35	Yeast two-hybrid	(1)
CBP	Yeast two-hybrid;Co-immunoprecipitation;GST-pull down	(4)
ARF	Yeast two-hybrid;Co-immunoprecipitation	(2)
RelA	Co-immunoprecipitation; Affinity precipitation;Chromatin immunoprecipitation	(5)
Chk1/2	Co-immunoprecipitation;GST-pull down	(6)
Maxer	Interactome database; Co-immunoprecipitation	(7)
DDRGK1,RCAD	Co-immunoprecipitation	(8)
NLBP/KIAA0776	Affinity chromatography; mass spectrometry; Co-immunoprecipitation;GST-pull down;	(9)
p38	Scansite analysis; Co-immunoprecipitation	(10)
PAK4	Co-immunoprecipitation;GST-pull down	(11)
γ-tubulin	mass spectrometry;Co-immunoprecipitationP; GST-pull down	(12)
Pre-S2 LHBs	Yeast two-hybrid; mammalian two-hybrid; Co-immunoprecipitation;GST-pull down;	(13)
p14^ARF^	Chromatin immunoprecipitation	(14)
Wip1, MDM2, HuR	Co-immunoprecipitation	(15)
P53,HDM2	Co-immunoprecipitation;GST-pull down	(15)
MCM6	Mass spectrometry;Co-immunoprecipitation	(16)
STAT3	RNA interference screen;Chromatin immunoprecipitation	(17)
HSF1	Co-immunoprecipitation;GST-pull down	(18)
ATG8	Immunoprecipitation coupled to mass spectrometry; GST-pull down;native mass spectrometry	(19)

By interacting with other proteins, CDK5RAP3 participates in the regulation of multiple cellular processes including cell cycle, apoptosis, cell invasion, signaling transduction, autophagy, UFMylation and endoplasmic reticulum (ER) stress. Furthermore, genetic studies in zebrafish show that CDK5RAP3 is necessary for epiboly and gastrulation, as well as normal dorsal-ventral patterning during early embryo development (3, 20). More recently, it has been reported that CDK5RAP3 is also required for mammalian tissue development such as liver and Paneth cell development (21, 22). CDK5RAP3 also has an intimate relationship with carcinogenesis and metastasis. CDK5RAP3 was initially considered as a putative tumor suppressor since it is found to be markedly reduced in 32% of primary head and neck squamous cell carcinoma (HNSCC), and inhibits cellular transformation and tumor growth *in vitro* and *in vivo* (5). Hereafter, it displays a tumor suppressor activity in hepatocellular carcinoma (23), gastric cancer (16, 24–27), and renal cancer (28) as well. In contrast, additional studies indicate that CDK5RAP3 appears to promote tumorigenesis because it is overexpressed in lung adenocarcinoma (29), hepatocellular carcinoma (11, 14), breast cancer (17) and cervical carcinoma (30), and has an oncogenic roles in these cancers. Hence, the roles of CDK5RAP3 in human cancers remain elusive and controversial. In addition, emerging research suggests that CDK5RAP3 may be involved in the regulation of hepatic, hematological and metabolic diseases due to the symptoms with liver degeneration, anemia, hemorrhage, and hypoglycemia, as well as impaired lipid metabolism and liver regeneration caused by its deficiency (21, 31). Consequently, CDK5RAP3 may be of importance for disease, especially tumor progression. In the following sections, we summarized the current advances in the molecular and functional basis of CDK5RAP3, and its implications for physiological and pathological conditions.

## The Gene Structure and Distribution Features of CDK5RAP3

**Figure 1:**
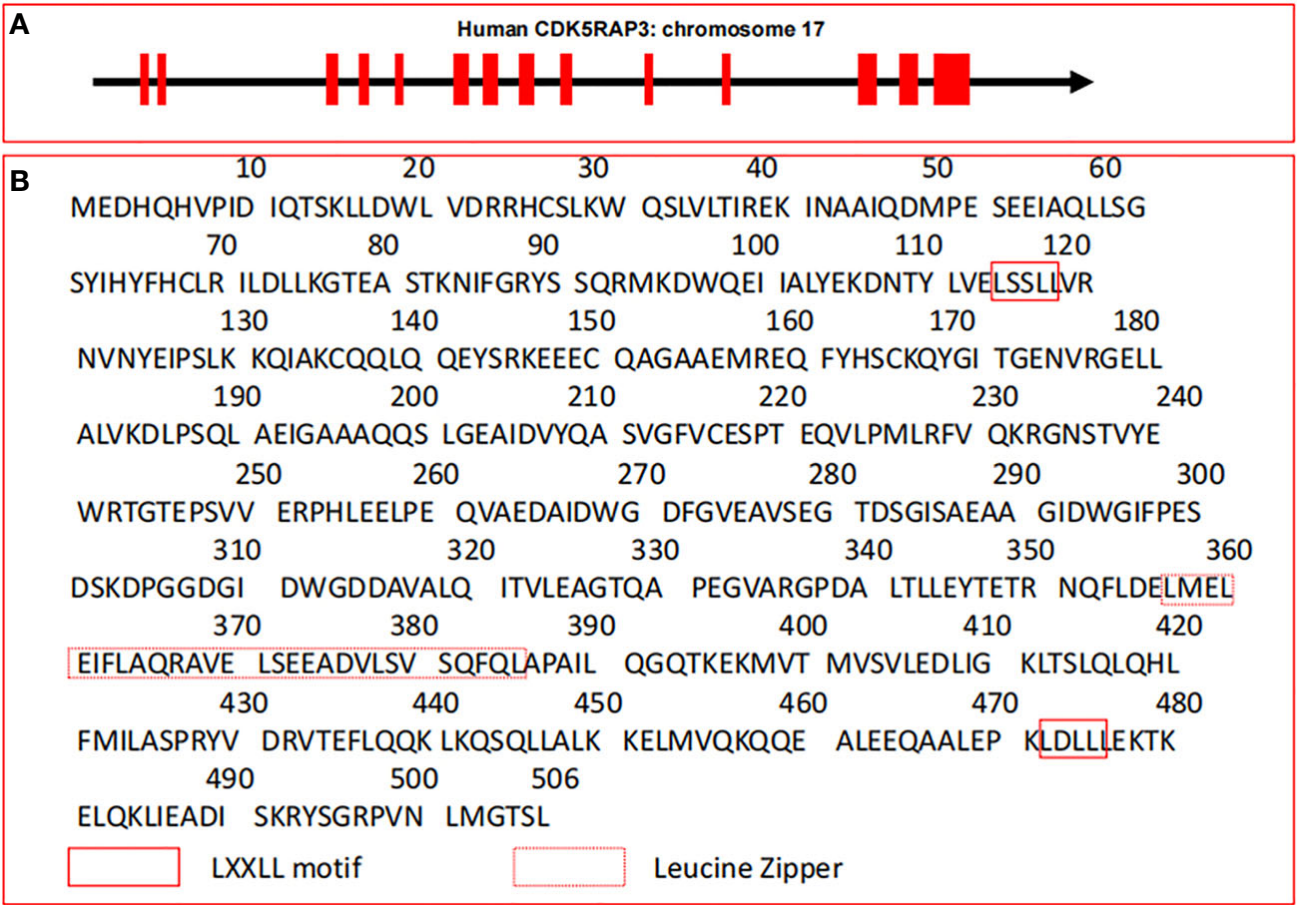
Gene structure and amino acid sequences of CDK5RAP3. **(A)** Gene structure of human CDK5RAP3 located on chromosome 17. Red filled boxes represent exons. **(B)** Canonical amino acid sequences of CDK5RAP3. The leucine zipper and the LXXLL motif of CDK5RAP3 are indicated.

Human canonical CDK5RAP3 gene with 14 exons is located at chromosome 17q21.32 (https://ncbi.nlm.nih.gov/gene/80279) and its gene ID is 80279 (**Figure 1A**). CDK5RAP3, also called as C53, was originally described as a CDK5 activator p35-binding protein (1). Whereafter, Wang and his group reported the finding of ARF-binding protein (LZAP) that is proved to be identical with CDK5RAP3 (2). As the expression of CDK5 regulatory subunit p35 is mainly in neurons of the central nervous system, its binding to CDK5RAP3 is not discovered to represent their primary functions, and presumably represents brain-specific regulation of these proteins (1).

Structurally, the canonical transcript of CDK5RAP3 gene includes a full-length cDNA of 1841 bp which encodes a protein with 506 amino acid residues (3, 6, 32, 33), and its molecular mass is 57 kDa (1). Intriguingly, CDK5RAP3 has no well-defined functional domains and described enzymatic activity except a small region of leucine zipper responsible for protein dimerization and two LXXLL motifs governing the linkage with transcription factors (2, 6, 10) (**Figure 1B**). These features are reminiscent of its interactions with various proteins and its functions as a transcriptional modulator. Particularly, CDK5RAP3 is found to be physically associated with multiple proteins to form a putative complex under certain circumstances (2, 8, 10). For instance, CDK5RAP3 is observed to combine with the animo-terminal region of ARF, and form a ternary complex with ARF and human double minute 2 (HDM2) in U2OS cells (2). One possible explanation for the action of these forming complexes is that CDK5RAP3 may serve to bring effector proteins together (10, 34). The other is that the association of CDK5RAP3 with its partners may increase their own protein stabilities (34). Thus, this phenomenon suggests that the formation of large molecular weight complexes may be pivotal to the function of CDK5RAP3 in some cases. Additionally, CDK5RAP3 lacks membrane-spanning motifs and putative signal peptide sequences, indicating that it may be an intracellular protein.

Evolutionally, CDK5RAP3 orthologues are highly conserved in vertebrates, invertebrates and plants but not in unicellular yeast and bacteria (3). The sequence similarity between human and murine CDK5RAP3 is more than 90%, and between zebrafish and human or murine CDK5RAP3 is over 80%, indicating that the conservation of CDK5RAP3 in genomic structure and function is rather high across mammalian species (3). In particular, based on the data of sequence alignment, the amino-terminal portion and carboxyl-terminal domain of CDK5RAP3 are more credibly conserved, indicating that these amino acids may represent vital functional regions of the CDK5RAP3 protein (3). Furthermore, it is reported that CDK5RAP3 has many different isoforms. For example, IC53 is an isoform of CDK5RAP3 from a human aorta cDNA library while IC53-2 is another isoform of CDK5RAP3 from a human placenta cDNA library (32, 35).

**Figure 2:**
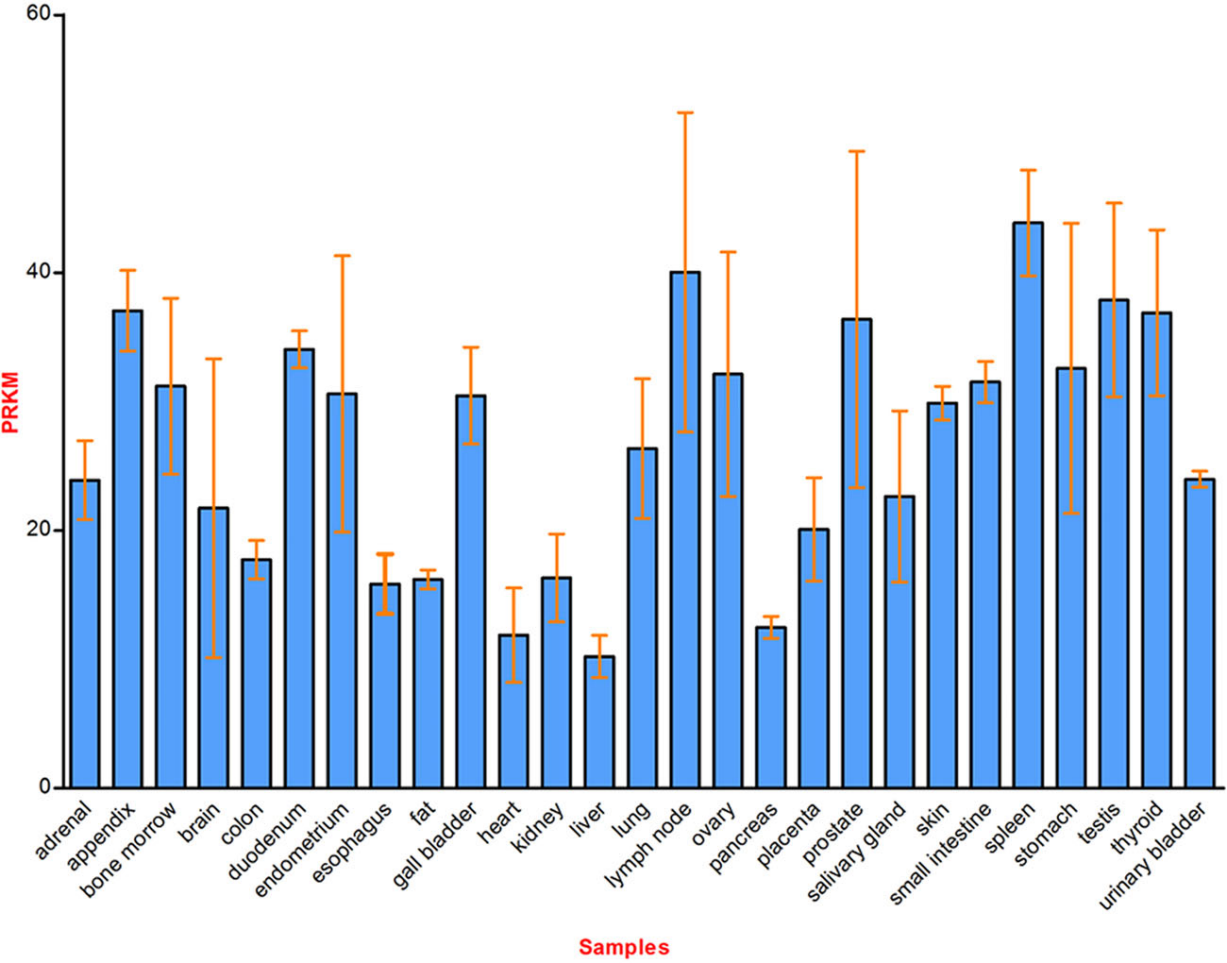
Schematic diagram of CDK5RAP3 mRNA expression in 27 different human tissues. Reads Per Kilobase Million (RPKM) value represents the corresponding mRNA abundance of CDK5RAP3 in different organs, and the data from www.ncbi.nlm.nih.gov/gene/80279.

**Figure 3:**
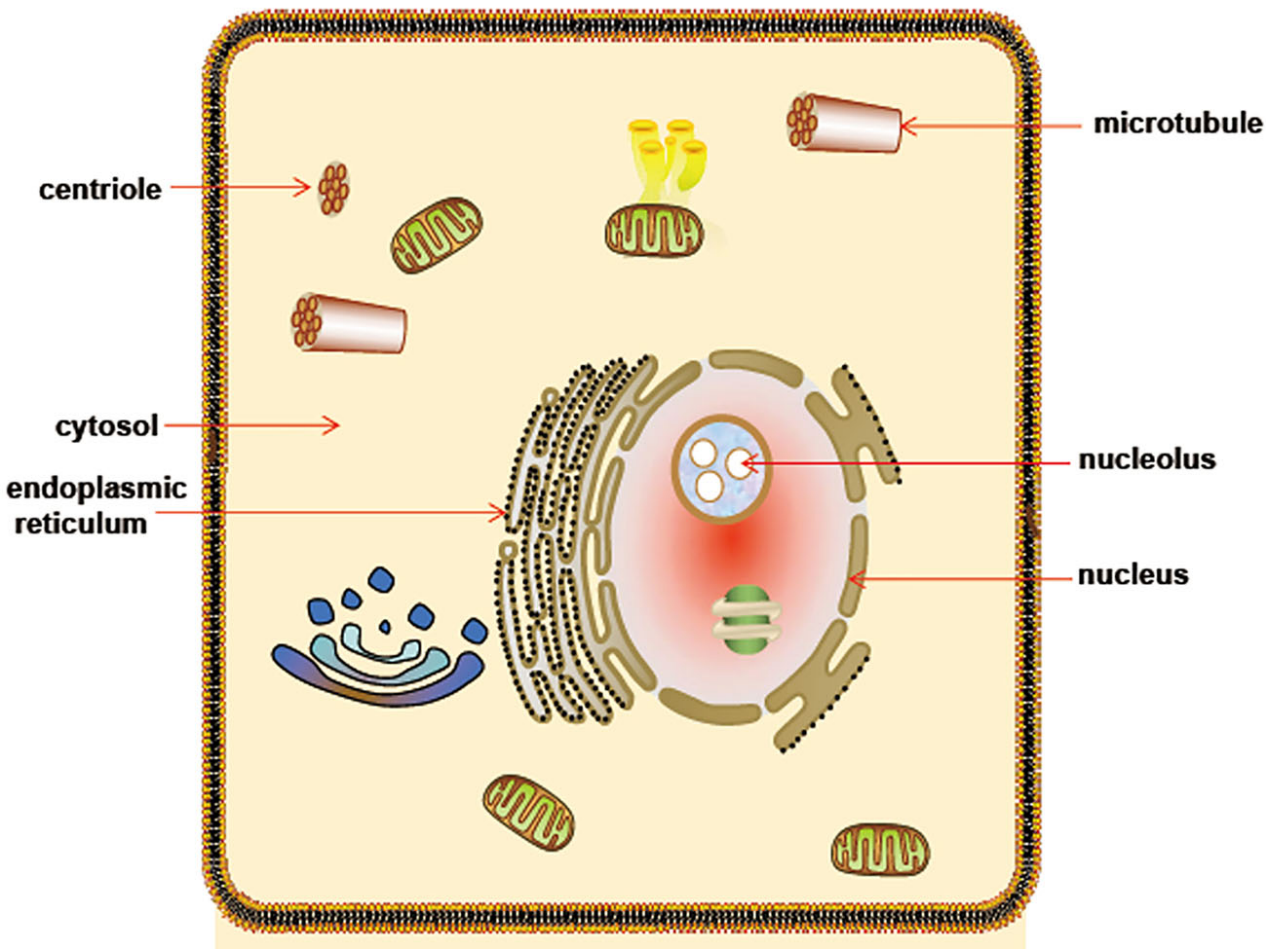
Schematic diagram of CDK5RAP3 subcellular localization. CDK5RAP3 is widely located at multiple subcellular compartments, involving cytosol, nucleus, nucleolus, microtubule, centriole and endoplasmic reticulum.

The data from northern blot shows that CDK5RAP3 is ubiquitously expressed in human tissues, such as the heart, brain, skeletal muscle, placenta, lung, liver, kidney, and pancreas (1). RNA-seq results reveal that CDK5RAP3 is widely expressed in 27 different human tissues (36) and **Figure 2** shows the mRNA abundance, i.e. Reads Per Kilobase Million (RPKM) value, of CDK5RAP3 in these organs. These results suggest that CDK5RAP3 may be a crucial gene in human tissues. Similarly, endogenous CDK5RAP3 is found to reside in multiple subcellular compartments (6, 12), including the cytosol, nucleus, nucleolus, centrosome, endoplasmic reticulum and microtubules (**Figure 3**). Interestingly enough, several studies have reported that the interaction of CDK5RAP3 with its partners can alter its subcellular distribution (2, 7, 11). For instance, Mak et al., revealed that the ectopic expression of CDK5RAP3 binding to p21-activated protein kinase 4 (PAK4) changes its localization, transferring from the cytoplasm and nucleus to the membrane periphery (11). Wang et al., found that co-expression of CDK5RAP3 with ARF results in the alternation of their subcellular distribution (2). Shiwaku et al., also confirmed that the knockdown of multiple α-helix protein located at ER (Maxer), an endoplasmic reticulum-associated protein, induces a shift of CDK5RAP3 from the cytoplasm to the nucleus (7). Therefore, these results strongly support the point that the translocation of CDK5RAP3 may be an important way to perform its function under certain circumstances. Furthermore, a recent study has reported that CDK5RAP3 may be a novel nucleoplasmic shuttle as it can interact with heat shock factor 1 (HSF1) and then inhibit HSF1 activation, thus affecting the nuclear and cytoplasmic distribution of HSF1 (18).

## Biological Functions of CDK5RAP3

The available data on CDK5RAP3 from PubMed and other databases shows that CDK5RAP3 is related to diverse cellular processes including cell cycle progression, apoptosis, cell adhesion/invasion, cellular signaling transduction, proteostasis, and so on. Nonetheless, the precise molecular mechanisms regulating its functions are far from being thoroughly understood.

### CDK5RAP3 as A Vital Controller of Cell Cycle and Apoptosis

It is well known that two of the most important proteins involved in the cell cycle control are CDKs and cyclins (37). A variety of factors are able to modulate the two proteins and influence their catalytic activities, thereby either hindering cell cycle progression for DNA repair or inducing cell death (38). Previous studies demonstrated that CDK5RAP3 overexpression can partially antagonize the role of the checkpoint kinase 1 and 2 (Chk1/2), a G2/M DNA damage checkpoint, and promote the CDK1/cyclinB1 complex activation, thereby enabling cancer cells susceptible to DNA damage agents and inducing apoptosis (6, 39). Moreover, they found that CDK5RAP3 can interact with Chk1/2 which in turn counteracts the activities of Chk1/2 and activates the CDK1/cyclin B1, thus accelerating the entry into mitosis both in unperturbed cell cycle progression and in DNA damage response (6). Additional studies found that CDK5RAP3 can interplay with diverse proteins to affect the G2/M checkpoint (12, 13). As an example, nucleolar γ-tubulin is found to associate with CDK5RAP3 and diminish CDK5RAP3-mediated activation of the CDK1/cyclinB1 during the G2/M DNA damage checkpoint (12). The binding of CDK5RAP3 to pre-S2 hepatitis B virus L proteins (pre-S2 LHBs) was also discovered to partially inhibit the checkpoint kinase Chk1 activity, further promoting CDK1 activation and mitotic entry (13). These studies markedly indicate that CDK5RAP3 may be a positive regulator of CDK1 activation through antagonizing the G2/M checkpoint kinases. In a zebrafish model, CDK5RAP3 loss in early embryonic cells is observed to result in a G2/M arrest, thereby inhibiting proliferation and increasing apoptosis, indicating that CDK5RAP3 is necessary for normal cell cycle (3).

p53 is extensively recognized as a cell cycle protein and a potent inducer of apoptosis (40, 41). Several studies have reported that CDK5RAP3 can lead to a G1 phase arrest and trigger apoptosis possibly in a p53-dependent manner (2, 15). Interestingly, CDK5RAP3 may regulate p53-mediated cell cycle and apoptosis through multiple pathways. Wang et al., found that CDK5RAP3 restores HDM2-directed p53 ubiquitination (Ub) but does not increase the p53 ubiquitinated degradation in the presence of ARF and HDM2, and ubiquitinated p53 is mainly located in the nucleus (2). Conversely, they observed that the association of CDK5RAP3 with ARF raises the p53 stability and increases p53 transcriptional activity, thus causing a G1 cell cycle arrest (2). Additionally, they discovered that CDK5RAP3 overexpression also results in a p53-dependent G1 arrest in the absence of ARF (2). Still, Wamsley et al., reported that CDK5RAP3 depletion reduces the p53 protein level regardless of its mutant status, thus protecting the cells with wild-type p53 from DNA damage-triggered apoptosis whereas sensitizing cells expressing mutant p53 to DNA damage (15). Meanwhile, they demonstrated that CDK5RAP3 activity toward p53 is independent of Wip and ARF, but is dependent on HDM2. Besides, Maxer, an ER membrane protein, is seen to interact with CDK5RAP3 and its deficiency translocates CDK5RAP3 from the cytoplasm to nucleus which inhibits the expression of cyclin D1 and then delays the G1/S transition (7, 42, 43).

It is generally documented that caspases have a core role in the initiation and execution of apoptosis, characterized by caspase-mediated cleavage of target proteins (44). Coincidentally, Jiang et al., demonstrated that ectopic expression of CDK5RAP3 leads to caspase-3 activation and promotes DNA damage-mediated apoptosis, suggesting that CDK5RAP3 may act as upstream of caspase activation during apoptosis (39). More interestingly, another group discovered that CDK5RAP3 is a caspase substrate, and caspase-mediated cleavage of CDK5RAP3 protein leads to abnormal microtubule bundling and rupture of the nuclear envelope during apoptosis (45). Accordingly, we propose a notion that there may be a feedback loop between apoptosis and CDK5RAP3. Nevertheless, how the feedback loop works needs to be further studied.

**Figure 4:**
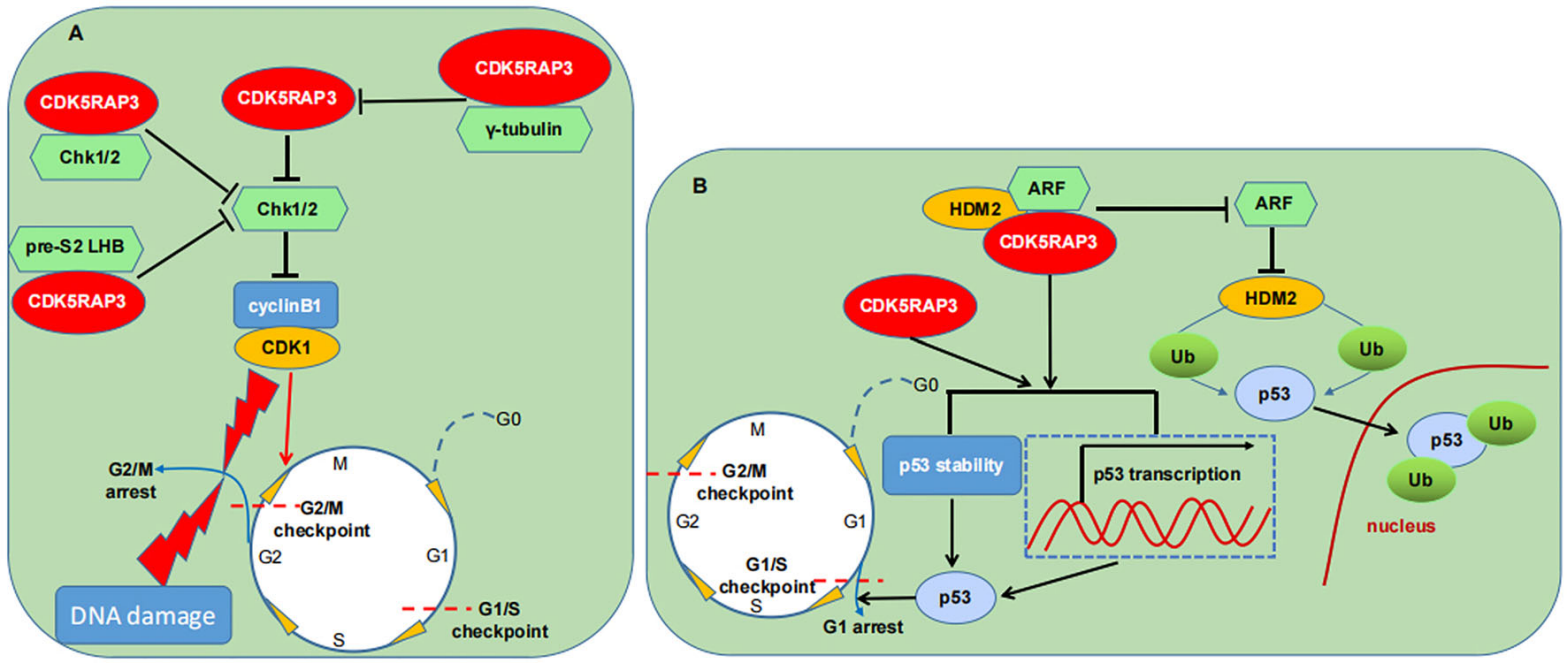
CDK5RAP3 and cell cycle machinery. **(A)** CDK5RAP3 affects the G2/M checkpoint through different pathways. **(B)** CDK5RAP3 affects the G1/S checkpoint in a p53-dependent manner. T-shaped arrow indicates inhibition; Standard-shaped arrow denotes promotion.

Combined, CDK5RAP3 is critical for cell cycle progression and apoptosis. Under different cellular environments, CDK5RAP3 affects cell cycle not only through delaying the exit from the G2 checkpoint (**Figure 4A**), but also through regulating the exit from the G1 checkpoint (**Figure 4B**), suggesting the CDK5RAP3-mediated cell cycle functioning may be diversified and complex. Meanwhile, the mechanisms of CDK5RAP3-directed apoptosis are rather intricate and need to be further elucidated.

### CDK5RAP3 as A Regulator of Cell Adhesion and Invasion

In a zebrafish model, CDK5RAP3 deficiency alters intercellular adhesion, likely leading to abrogation of epiboly, suggesting that CDK5RAP3 may play a role in cell adhesion and progress of epiboly (3). CDK5RAP3 is also considered as a regulator of cell invasion. Depletion of CDK5RAP3 is found to promote cell invasion and matrix metalloproteinase (MMP)-9 expression through activating nuclear factor (NF)-κB pathway in a U2OS cell model (2). The loss of novel LZAP-binding protein (NLBP), a binding partner of CDK5RAP3, can result in loss of CDK5RAP3, thereby enhancing cell invasion and NF-κB activation (8, 9). Given that cell adhesion and invasion are essential properties of tumor metastasis in malignancies (46, 47), CDK5RAP3 is also an important participant in tumor metastasis (11). Nevertheless, its exact function in cancer cell invasion remains conflicting. A group demonstrated that CDK5RAP3 promotes the cell migration and invasiveness in HCC cell lines SMMC-7721 and HepG2 (11, 14). However, another group discovered that CDK5RAP3 inhibits the migration and invasion in HCC cell lines HepG2 and sk-Hep1 (23). Interestingly enough, both studies apply the same cell line to *in vitro* experiments and performed immunohistological staining of CDK5RAP3 in tens of patients. Thus, further work remains to explore the precise function of CDK5RAP3 in cell invasion and its possible mechanism.

### Signaling Pathways Regulated by CDK5RAP3

**Figure 5:**
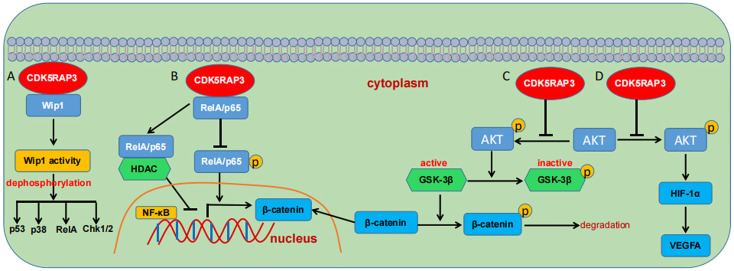
Schematic overview of the signaling pathways regulated by CDK5RAP3. **(A)** CDK5RAP3 binding to Wip1 promotes its activity and augments its dephosphorylation to downstream substrates. **(B)** CDK5RAP3 negatively regulates the NF-κB signaling pathway. **(C)** CDK5RAP3 regulates the Wnt/β-catenin signaling pathway. **(D)** CDK5RAP3 modulates the AKT/HIF1α/VEGFA signaling pathway. T-shaped arrow indicates inhibition; Standard-shaped arrow denotes promotion.

Accumulating evidence indicates that CDK5RAP3 has been implicated in governing cellular signal transduction including the p53 (2, 15), NF-κB (5, 8, 9, 48), Wnt (20, 24, 25), wild-type p53-induced phosphatase 1 (Wip 1) (10, 33), AKT (27, 30) and signal transducer and activator of transcription 3 (STAT3) signaling cascades (17). As previously described, CDK5RAP3 positively regulates p53 activity either in the presence or absence of ARF or independently of p53 mutation state (2, 15). Again, one group displayed that CDK5RAP3 is a novel binding partner of Wip 1 and stimulates its phosphatase activity, subsequently augmenting the dephosphorylation of its substrates such as RelA, p38, Chk1/2, p53 (15) (**Figure 5A**). CDK5RAP3 is also documented to negatively modulate the NF-κB signaling by connecting directly with RelA (p65), impairing its phosphorylation at serine 536 and promoting its binding to histone deacetylase (HDAC), thereby inhibiting NF-κB transcriptional activity (5) (**Figure 5B**). Additional data witnesses that CDK5RAP3 has a close association with AKT and Wnt/β-catenin signaling (**Figure 5C**). CDK5RAP3 loss is found to cause an increase in phosphorylated glycogen synthesis kinase-3β (GSK-3β), which in turn leads to a reduction in phosphorylated β-catenin and its nuclear accumulation (20, 24). The study from Zheng et al., also showed that CDK5RAP3 suppresses the inhibitory phosphorylation of GSK3β *via* inhibiting AKT phosphorylation in gastric cancer (25). Moreover, their group demonstrated that CDK5RAP3 modulates AKT signaling in both gastric neuroendocrine and cervical carcinoma (27, 30). CDK5RAP3 also negatively regulates AKT/hypoxia inducible factor-1α (HIF-1α)/vascular endothelial growth factor A (VEGFA) pathway in gastric neuroendocrine carcinoma (GNEC) (**Figure 5D**). Specifically, CDK5RAP3 inhibits the phosphorylation of AKT, which decreases the expression of HIF-1α and its downstream VEGFA (27).

In the light of the above-stated findings, there is no doubt that CDK5RAP3 is an important player in signal transduction. However, due to the presence of the crosstalk between signaling pathways and the diversity of its target proteins, the extent and details of CDK5RAP3-mediated signaling transduction remain to be thoroughly studied.

### CDK5RAP3 as a Potential Participant in Autophagy

Autophagy is a key intracellular degradation process during which eukaryotic cells clear up harmful or unwanted cytoplasmic contents to maintain cellular homeostasis (49–51). Current research demonstrates that many factors are involved in modulating this process (19, 28, 52–54). Among them, CDK5RAP3 is reported to regulate autophagy in a renal cancer model (28). Knockdown of CDK5RAP3 in the human renal cell line Caki-1 cells results in a decrease of an autophagy-related protein microtubule associated protein 1 light chain 3 (LC3) conversion, whereas its overexpression in the human renal cell line 769-P cells leads to an increase of the LC3-II level. These data indicate that CDK5RAP3 may discrepantly regulate autophagy in different cells. More recently, CDK5RAP3 is identified as an emerging regulator of selective autophagy in ER, namely ER-phagy (19, 54). Stephani et al., demonstrated that CDK5RAP3 interacts with autophagy associated gene (ATG) 8, the non-mammalian homologue of LC3, through non-canonical ATG8 interacting motifs in plant and mammalian models (19). And they uncovered that CDK5RAP3 is activated by ribosome stalling during co-translational protein translocation. Additionally, their results showed that CDK5RAP3-mediated autophagy can be activated by phosphate starvation, not by carbon or nitrogen starvation (19). Nevertheless, Liang et al. showed that CDK5RAP3 mutant does not affect ER-phagy (55). Difference between Stephani’s and Liang’s findings shows that the association of CDK5RAP3 with autophagy depends on cell-type (19). To date, the reports on the relationship between CDK5RAP3 and autophagy are rather limited. Further investigation is required to explain the exact role of CDK5RAP3 in autophagy and its working mechanisms.

### CDK5RAP3 as a Satellite Component of the UFMylation System

The UFMylation is referred to as a process of post-translational modification that is orchestrated by the sequential action of ubiquitin fold modifier 1(UFM1) and three enzyme classes, namely the E1 ubiquitin activating enzyme 5 (UBA5), E2 UFM1-conjugase 1 (UFC1) and E3 UFM1-conjugase 1(UFL1) (56, 57). The detailed knowledge on UFMylation cascade has be elaborated in published reviews (34, 58).

Increasing data supports the view that CDK5RAP3 is a candidate satellite component in the UFM1 conjugation system (7–9, 59–61). Firstly, CDK5RAP3 is confirmed to interact with several core components of the UFMylation system such as UFM1 and E3 ligase UFL1, and its adaptor protein DDRGK domain containing protein 1 (DDRGK1). In particular, previous literatures strongly suggested that UFL1, together with DDRGK1 and CDK5RAP3, may form a protein complex that affects the UFMylation of substrates (8, 19, 21, 34, 55). Secondly, CDK5RAP3 is found to take part in the UFMylation of UFM1 targets under certain circumstances (60) and play an essential role in poly-UFMylation (62). Thirdly, the three proteins CDK5RAP3, UFL1 and DDRGK1 are of high similarity in species and tissue expression, and subcellular localization (8). Furthermore, the intervention of one protein affects the expression level of another one (8). Finally, these protein-directed functions in many scenarios are extremely similar such as the regulation of NF-κB signaling, cell invasion, hematopoiesis and ER stress (8, 19, 21, 34, 63, 64). As such, it is possible that the UFMylation machinery is one of the working modes of CDK5RAP3-mediated biological effects. Notwithstanding, CDK5RAP3, as stated earlier, can affect the phosphatase activity rather than UFMylation of its targets in many instances. Therefore, the mechanistic link between CDK5RAP3 and the UFMylated cascade requires further investigation.

### CDK5RAP3 as a Pivotal Modulator During ER Homeostasis

The endoplasmic reticulum (ER), an important network of membranes, is regarded as the core machinery in protein biosynthesis, folding, maturation, quality control and degradation (65, 66). To ensure the quality of proteins, the ER is dynamically regulated by an intricate signal transduction system related to the secretory pathway. The perturbation of the ER homeostasis, namely ER stress may lead to activation of the unfolded protein responses (UPR) to reestablish cellular homeostasis (67). The regulation of the UPR pathway is controlled largely by three key sensors, that is the inositol-requiring enzyme 1 (IRE1) α, pancreatic endoplasmic reticulum kinase (PERK), and activating transcription factor 6 (ATF6) (68, 69). All the three sensors are usually combined with the ER resident chaperones like glucose-regulated protein 78 (GRP78)/binding immunoglobulin protein (Bip) through their ER luminal domains which keeps them in an inactive state. And when Bip binds to accumulating misfolded/unfolded proteins, it can release the three sensors and then activate the UPR cascade during ER stress. If the UPR strategy is unable to dispose of ER stress, the ER-associated degradation (ERAD) will be initiated to resolve the unfolded or misfolded proteins either by a ubiquitin-proteasome or by an autophagy-lysosome dependent mechanism, and molecular chaperones will be upregulated to promote the proper protein folding (70).

Dozens of studies have shown a connection between CDK5RAP3 and ER homeostasis. As stated above, DDRGK1, a well-documented ER membrane protein, is a physically binding partner of CDK5RAP3. As such, CDK5RAP3 may relocate to the ER under the recruitment of DDRGK1 where it regulates ER-related proteins (8, 34, 62). As an example, DDRGK1 is proved to recruit the localization of UFL1 and CDK5RAP3 to ER, and then promote the UFMylation of ribosomal protein L26 (RPL26) at the lysine 134. Subsequently, UFMylated RPL26 mediates the degradation of ER sheets and a quality control factor ribophorin1 glycosylating faulty ER proteins (55, 62, 71). Again, CDK5RAP3 is a satellite component of the UFMylation cascade as well (34). The UFMylation system is essential to maintain cell homeostasis including ER homeostasis (72–74). For example, the increased activity of the UPR cascades is observed in mice with UFMylation impairment, representing by the activation of the IRE1 and PERK pathway, concomitantly with upregulation of their downstream spliced X-box binding protein 1(Xbp1s) and phosphorylated eukaryotic translation initiation factor 2 alpha (eIF2α) (75). On the other hand, the induction of ER stress is observed to be accompanied by the upregulation of UFMylation cascades. An example is that UFM1 is found to be transcriptionally upregulated in response to ER stress in an ischemic heart disease mice model (76). Importantly, the substrate spectrum of UFMylation has been recently expanded to ribosomal proteins such as 80S ribosome, RPL26, ribosomal subunits, namely uS3, uS10 and uL16 (62, 77, 78). Pleasingly, two or three studies have demonstrated that CDK5RAP3 directly affects the ER stress (21, 22, 31). In a hepatocyte-specific deletion of CDK5RAP3 model, CDK5RAP3 loss results in the ER stress, concomitantly with activation of IRE1α and PERK signaling pathways (21). Similarly, CDK5RAP3 deficiency caused activation of the UPR, especially the IRE1α-Xbp-1 branch, in an intestinal epithelial cell-specific knockout mouse model (22).

Overall, The UFMylation conjugation is a vital player in maintaining ER homeostasis. The current advances on the UFMylation system and ER network can be consulted in several published reviews (34, 58, 72). Remarkably, CDK5RAP3, both as a UFMylation component and as a candidate ER-phagy player, may play a master role in ER homeostasis. Nonetheless, as a multifaceted protein, how CDK5RAP3 combines the UFMylation pathway with autophagy and ER stress remains unresolved.

## Implication of CDK5RAP3 for Development

### CDK5RAP3 in Embryogenesis

Data from zebrafish and mice models has shown that CDK5RAP3 is essential for embryonic development. In a zebrafish model, CDK5RAP3 ablation is found to not only fail to initiate epiboly (3), which is the first morphogenetic movement of zebrafish embryo during the gastrulation stage (79), but also disturb normal dorsal-ventral patterning during early development (20). Consistent with these observations, in a mouse model, CDK5RAP3 deficiency is demonstrated to be lethal to the embryo (3, 21, 22). For instance, CDK5RAP3 loss in mice causes severe liver hypoplasia and subsequent embryonic lethality (21). Interestingly, there is a discrepancy in these reports regarding the timing of embryonic lethality. One group reported that CDK5RAP3 deletion mice die from embryonic day 16.5 onwards (21), whereas others found that the timing of mice death is earlier, either after embryonic day 6.5 or after embryonic day 8.5 (3, 22). And the reason for this inconformity remains to be understood.

### CDK5RAP3 in Tissue Development

Many observations have indicated that the core components of the UFMylation system UFM1, UBA5, and UFL1 have a direct association with multiple cell development (63, 80–82). Akin to these key components, CDK5RAP3 is recently reported to be involved in a couple of tissue development. Yang et al., reported that CDK5RAP3 is essential for postnatal hepatocyte growth, proliferation and function maturation (21). Hepatocyte-specific CDK5RAP3 deficient mice also display serious hypoglycemia and lipid metabolism disorders which results in post-weaning lethality (21). Quintero et al., showed that intestinal epithelial cell-specific deletion of CDK5RAP3 causes complete depletion of Paneth cells, and its deletion in mature Paneth cells also results in the loss and abnormality of Paneth cells, indicating CDK5RAP3 is necessary to Paneth cell development and maturation (22). Moreover, both of them propose a possible working mechanism that CDK5RAP3 may regulate tissue development *via* the UFMylation pathway (21, 22). The most immediate evidence may be that CDK5RAP3 knockout in both hepatocytes and intestinal epithelial cells directly alters the UFMylation cascade. Reminiscent of the CDK5RAP3/UFL1/DDRGK1 complex in the ER, this complex may recruit specific UFMylated substrates to maintain the ER homeostasis in these cells (21, 22). However, given that CDK5RAP3 is a multi-faceted protein, whether it acts by other mechanisms in these tissues needs to be further investigated.

## CDK5RAP3 and Disease

### CDK5RAP3 as a Tumor Suppressor or Promotor?

Numerous studies have revealed the potential inhibitory role of the CDK5RAP3 in the context of various cancers. In the HNSCC, Wang et al., found that CDK5RAP3 protein levels are significantly reduced in more than 30% of primary human HNSCC which is negatively correlated with expression of NF-κB target genes, including interleukin-8 (IL-8) and IκBα, and its depletion induces primary cell transformation (5). Additionally, they discovered that decreased CDK5RAP3 level promotes xenograft tumor growth and blood vessel density in a HeLa cell-induced xenograft mice model, but also enhances NF-κB-dependent cellular invasion and MMP9 expression in a U2OS cell model (5). In the HCC, CDK5RAP3 expression is markedly decreased in the HCC tissues and cell lines, and its decreased expression is strongly associated with tumor size, histopathological classification, serum α-fetoprotein and poor prognosis (23). They also found that CDK5RAP3 expression suppresses HCC cell proliferation, migration, invasion and xenograft tumor growth, and induces apoptosis (23). In the stomach, CDK5RAP3 also functions as a tumor suppressor both in gastric cancer and in GNEC. The expression level of CDK5RAP3 is significantly reduced both in gastric cancer and in GNEC tissues which is correlated with TNM stage, poor prognosis, invasion depth, and lymph node metastasis (16, 24–27, 83). In addition, the ectopic expression of CDK5RAP3 suppresses cell proliferation, migration, invasion, angiogenesis, xenograft growth, and cancer stem-like cells (CSCs) phenotype, and epithelial-mesenchymal transition (16, 24–27, 83). Mechanically, CDK5RAP3 is reported to block the inhibitory phosphorylation of GSK-3β *via* repressing AKT activation, and then increase β-catenin phosphorylation for its degradation, thereby suppressing its nuclear translocation (24, 25). CDK5RAP3 is also confirmed to interact with minichromosome maintenance 6 (MCM6) and hinder its translocation into the nucleus, resulting in the inhibition of gastric cancer cell proliferation (16). Additionally, CDK5RAP3 is found to inhibit the phosphorylation of AKT and then reduce the expressions of HIF-1α and VEGFA, thereby suppressing angiogenesis in GNEC (27). The extracellular signal-regulated kinase (ERK) signaling is demonstrated to be an upstream modulator that restrains the expression of CDK5RAP3 in gastric CSCs (83). In renal cancer, CDK5RAP3 is downregulated in renal cancer tissues and participates in autophagy regulation in renal cancer cell lines (28). In summary, these results suggest that CDK5RAP3 may function as a tumor suppressor for these cancers (**Table 2**).

**Table 2:** Expression levels of CDK5RAP3 and its implications for different tumors.

Tumor type	Expression	Malignant cellular behavior	Potential mechanism	Clinical features	References
HNSCC	down	Inhibit transformation	NF-κB signaling	–	(5)
HCC	down	Inhibit proliferation, migration, invasion, and xenograft tumor growth; induce apoptosis	–	Tumor size, differentiation, serum AFP, prognosis	(23)
Gastric cancer	down	Inhibit proliferation, migration, invasion, and xenograft tumor growth	Wnt/β-catenin signaling	prognosis	(24–26)
Gastric cancer	down	Inhibit proliferation and migration	MCM6 translocation	TNM stage, prognosis	(16)
Gastric cancer	down	Inhibit self-renewal, invasion, EMT, and CD44 expression; promote chemoresistance	ERK1/2 signaling	Lymph node metastasis, N stage, prognosis	(83)
GNEC	down	Inhibit endothelial cell migration, tube formation, xenograft tumor growth and angiogenesis	AKT/HIF-1α/VEGFA signaling	Invasion depth, lymph node metastasis, TNM stage	(27)
Renal cancer	down	Inhibit cell viability; autophagy	LC3 conversion in Caki-1 cell; LC3 expression in 769-P cell	–	(28)
Lung adenocarcinoma	up	–	–	–	(29)
HCC	up	Promote proliferation, migration, invasiveness, and xenograft tumor growth	PAK4 activation	Tumor microsatellite formation, differentiation, extrahepatic metastasis	(11)
HCC	up	Promote migration and invasiveness	p14^ARF^ transcription activity	–	(14)
Breast cancer	up	Promote clonogenesis and migration	STAT3 transcription activity	–	(17)
Cervical carcinoma	up	Promote proliferation, invasion, migration, EMT, and xenograft tumor growth	AKT pathway	_	(30)

On the contrary, other studies have suggested that CDK5RAP3 may play a pro-tumorigenesis role in a couple of carcinomas. First, CDK5RAP3 is proved to be overexpressed in lung adenocarcinoma tissues (29). Second, CDK5RAP3 is also frequently upregulated in human HCC tissues and cell lines, and this overexpression is closely related to more aggressive phenotype, including more tumor microsatellite formation and extrahepatic metastasis, and poor differentiation (11). Both gain-of-function and loss-of-function experiments indicate that CDK5RAP3 can increase cell proliferation, migration, invasiveness and xenograft tumor growth in HCC cell lines (11, 14). Third, the abnormalities of CDK5RAP3 gene, including point mutations, deletions and insertions, are found to be infrequent or even absent, whereas the most common alteration of this gene is increased in copy number and gene overexpression by analyzing the COSMIC database (17). CDK5RAP3 is also observed to enhance the clonogenesis and migration in breast cancer cells (17). Fourth, CDK5RAP3 is markedly overexpressed in cervical carcinoma based on the data from several online databases and enhances the proliferation and tumorigenicity in cervical carcinoma cells (30). In addition, several CDK5RAP3 isoforms are observed to show an oncogenic phenotype (84, 85). For instance, IC53, a kind of CDK5RAP3 isoforms, is demonstrated to be positively correlated with the grade and invasion depth in colon adenocarcinoma, and enhance cell proliferation, migration, adhesion, and tumorigenicity of human colon cancer cell lines (84). Another CDK5RAP3 isoform IC53d is shown to be up-regulated in human gastric cancer tissues and its overexpression promotes tumorigenesis *in vitro* and *in vivo* (85). Several potential mechanisms have been proposed to elucidate the oncogenic function of CDK5RAP3 as well. One possible molecular mechanism is that CDK5RAP3 firstly binds to p21-activated protein kinase 4 (PAK4) and then this binding elicits the activation of PAK4 to promote HCC metastasis (11). Another potential mechanism is that CDK5RAP3 directly combines with the p14^ARF^ promoter and then represses its transcription to downregulate the expression of p14^ARF^, thereby promoting HCC metastasis (14). Meanwhile, they pointed out that both the overall integrity and nuclear localization of CDK5RAP3 are important for its repressor activity for p14^ARF^ (14). Besides, CDK5RAP3 in breast cancer cells is reported to positively regulates the transcriptional activity of signal transducer and activator of transcription 3 (STAT3), an oncogenic transcription factor, to trigger its tumorigenic phenotypes (17). Thus, all these studies indicate that CDK5RAP3 may also act as an oncogene for these cancers (**Table 2**).

Taken together, these data indicate that CDK5RAP3 has a marked relationship with human carcinoma but its precise function seems to vary greatly depending on tumor types, tumor stages or experimental condition. One of possible reasons is its diversity in interacting proteins and signaling transduction. Consequently, the molecular mechanisms of CDK5RAP3 acting as a tumor suppressor or promotor appear to be complicated and diverse. Furthermore, UFL1, a binding partner of CDK5RAP3, is also identified as a putative tumor suppressor in hepatocarcinoma or a potential oncogene in lung adenocarcinoma (9, 86). This opposite consequence may depend on UFM1’s substrate availability in different cancer types (34). Thus, further mechanistic investigations will be established to explain whether CDK5RAP3 performs its functions really by different ways in different types of cells and tissues or by a unified molecular mechanism.

### CDK5RAP3 as a Potential Cause of Other Diseases

Genetic studies have demonstrated the protective roles of the UFMylation conjugation system in multiple pathological disorders. For instance, cardiac-specific UFL1 knockout mice develop age-dependent cardiomyopathy and heart failure, manifested with elevated cardiac fetal gene expression, increased fibrosis and impaired cardiac contractility (64). Inducible deletion of UFL1 or UFBP1 (also known as DDRGK1) in adult mice causes severe anemia (63, 80). UFBP1-deficient mice are shown to be susceptible to experimentally induced colitis (87). In the pancreas, overexpression of UFM1 and UFBP1 protects pancreatic β cells from ER stress-induced apoptosis (59). The combination of alcohol consumption and UFL1 depletion leads to increased caspase 3 and trypsin activation in pancreatic acinar cells (88).

Given that it is considered as a vital satellite component of the UFMylation pathway, CDK5RAP3 may be involved in other diseases besides cancer. Recent studies have revealed that CDK5RAP3 knockout mice display liver degeneration, regeneration, anemia, and hemorrhage, as well as metabolic disorders such as hypoglycemia and impaired lipid metabolism, indicating that CDK5RAP3 may be associated with hepatic, hematological and metabolic diseases (21, 31). Moreover, the loss of CDK5RAP3 alters the UFMylation profile in liver cells (21). Additionally, in a diabetic mouse model, CDK5RAP3 expression is recently found to be downregulated which supports its involvement in metabolic diseases (48). More recently, Quintero et al., found that intestinal epithelial cell-specific knockout of CDK5RAP3 displays increased susceptibility to experimentally induced colitis in mice (22). They also found that CDK5RAP3 deletion results in defective UFMylation pathway and activation of UPR (22). As such, the potential mechanism underlying CDK5RAP3’s function in these diseases is partially mediated by the UFMylation pathway.

## Conclusion

Collectively, we overview the current research on CDK5RAP3, and highlight the following points. First, CDK5RAP3 structurally lacks well-defined functional domains and described enzymatic activity, but has a domain of leucine zipper and two LXXLL motifs, indicating that CDK5RAP3 executes its functions mainly by two central modes, i.e. protein-protein interaction and transcriptional regulation. Second, CDK5RAP3 shows evolutionally conserved in multicellular species, and stably expresses in various tissues and their subcellular compartments. Third, CDK5RAP3 can interact with a range of proteins and is a truly multifunctional molecule. Its functions cover most of biological processes, varying from cell survival, differentiation, adhesion/invasion, signal transduction, protein post-translational modification and cellular homeostasis. Furthermore, during these biological processes, CDK5RAP3 as a scaffold may regulate the activity, transcription or UFMylation of its target proteins. Fourth, CDK5RAP3 is essential for embryogenesis and mammalian development. Finally, CDK5RAP3 may be involved in several pathological disorders, especially tumorigenesis. By this token, These results provide useful information for further investigations on its involvement in diseases.

Admittedly, there are some unexploited, puzzling and conflicting questions about CDK5RAP3 to be unraveled. For instance, while a large number of CDK5RAP3 binding proteins have been identified, many questions regarding mechanistic understanding remain elusive. Although CDK5RAP3 has been demonstrated to be implicated in the regulation of protein phosphorylation and UFMylation, the preference of its target proteins for protein modifications is still obscure. Although some of CDK5RAP3-regulated signaling pathways have been described, whether there are additional pathways or whether there are crosstalks among these pathways remains unclear. In addition, based on the present reports, the function of CDK5RAP3 in tumor is depending on cell or tissue type. In most cases, CDK5RAP3 acts as a tumor suppressor, inhibiting the cell proliferation, migration and invasion, as well as inducing apoptosis. Rather, several studies support the viewpoint that CDK5RAP3 is a tumor promotor. These results propose a challenging question to CDK5RAP3 as a target for the treatment of tumors. Thus, some strategies remain to be explored to achieve more accurate treatment. Again, as a multifaceted protein, whether CDK5RAP3 is involved in more diseases beyond cancer is also a unresolved problem. But any way, the present findings have provided an opportunity for shedding enormous insight into physiological and pathological actions of CDK5RAP3 and for further interrogating its functions in cancer and other diseases.

## Author Contributions

LS, JL, and YH wrote the manuscript. SR and ZY designed the Figure and Table, and revised the manuscript. All authors read and approved the manuscript.

## Funding

This work was supported by grants from the National Natural Science Foundation of China (grantnos.81660163, YH) and Innovation and Entrepreneurship Training Program Project of Nanchang University (grantnos.2020CX280, ZY).

## Conflict of Interest

The authors declare that the research was conducted in the absence of any commercial or financial relationships that could be construed as a potential conflict of interest.

## Publisher’s Note

All claims expressed in this article are solely those of the authors and do not necessarily represent those of their affiliated organizations, or those of the publisher, the editors and the reviewers. Any product that may be evaluated in this article, or claim that may be made by its manufacturer, is not guaranteed or endorsed by the publisher.
